# Supplementary material for: Mechanism of Brevibacillus brevis strain TR-4 against leaf disease of Photinia×fraseri Dress
Source: PeerJ. 2024 Jun 25;12:e17568. doi: 10.7717/peerj.17568 (PMC11212632; doi:10.7717/peerj.17568)
Supplement: Supplemental Information 1 [file peerj-12-17568-s001.docx]

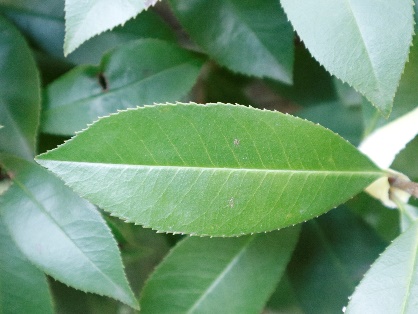

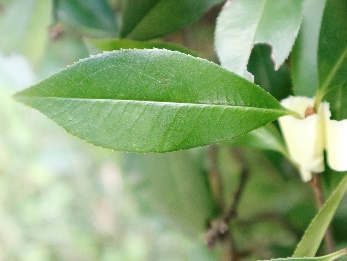

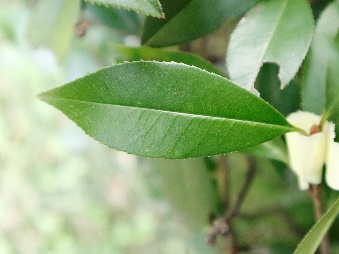

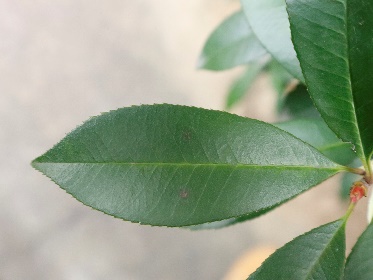

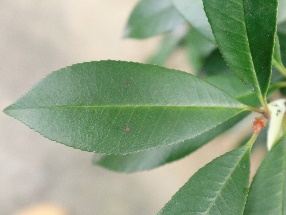

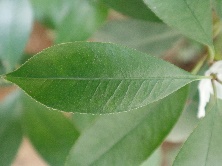


Leaf protection and pathogen inhibition experiment day 3


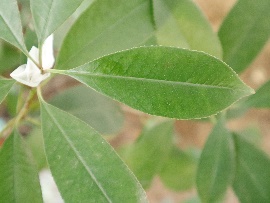

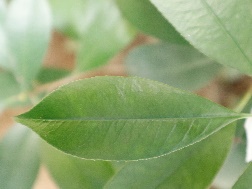

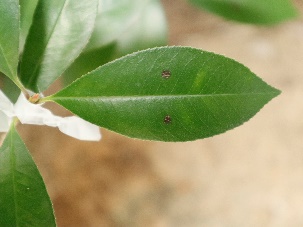

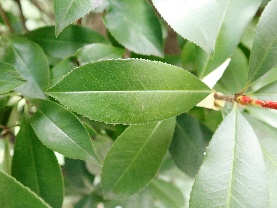

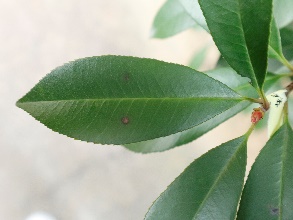


Leaf protection and pathogen inhibition experiment day 5


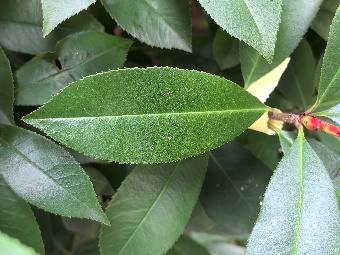

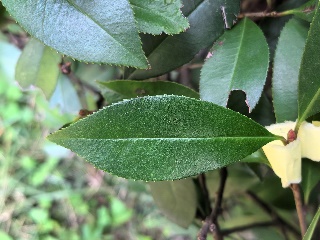

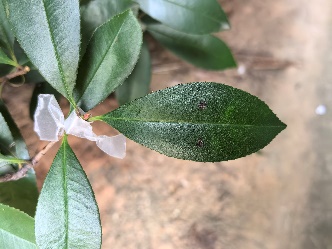

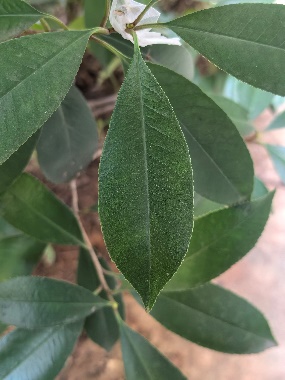


Leaf protection and pathogen inhibition experiment day 7


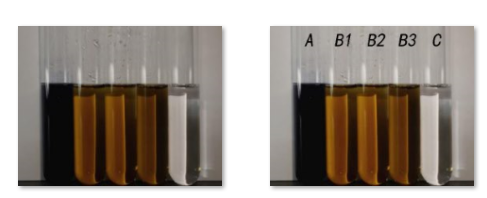


Ferrophilin test







Transmission electron microscope result


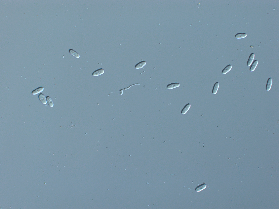

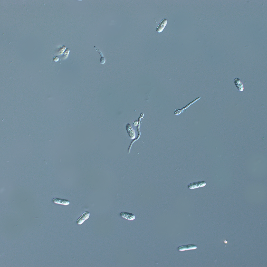

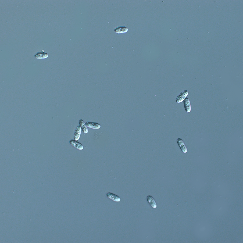

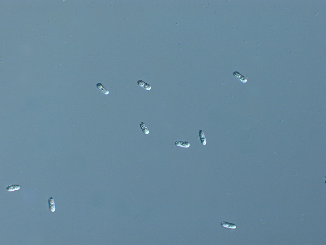


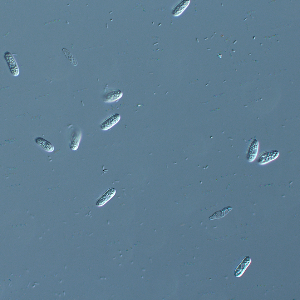

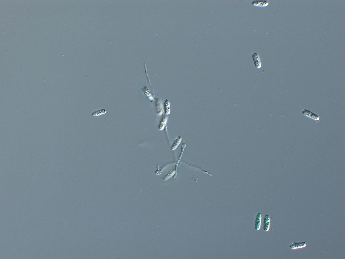

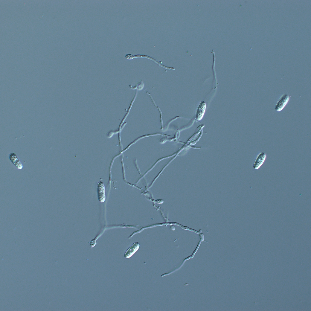

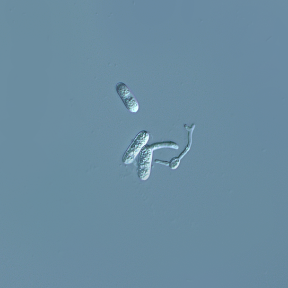


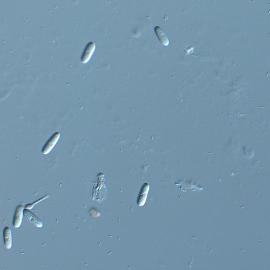

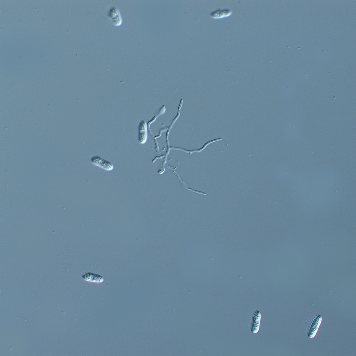

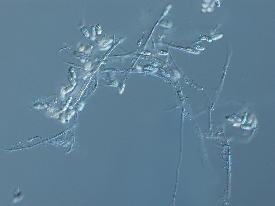

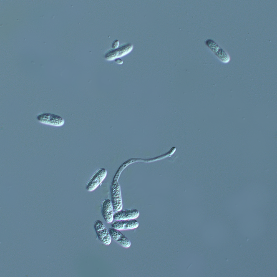


Spore germination microscopy


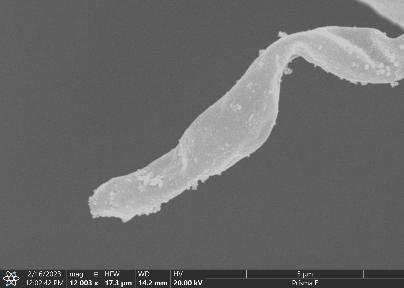

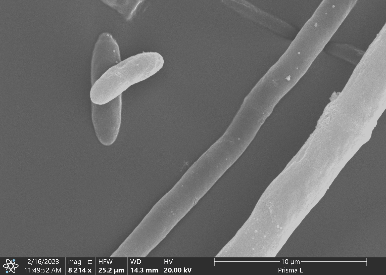


SEM observation

TR-4 16s sequence

>TR-4 ACTACGTGTGCTATAATGCAGTCGAGCGAGGCTCTTCGGACGCTAGCGGCGGACGGGTGAGTAACACGTAGGCAACCTGCCTCTCAGACTGGGATAACATAGGGAAACTTATGCTAATACCGGATAGGTTTTTGGATCGCATGATTCGAAAAGAAAAGATGGCTTCGGCTATCACTGGGAGATGGGCCTGCGGCGCATTAGCTAGTTGGTGGGGTAACGGCCTACCAAGGCGACGATGCGTAGCCGACCTGAGAGGGTGACCGGCCACACTGGGACTGAGACACGGCCCAGACTCCTACGGGAGGCAGCAGTAGGGAATTTTCCACAATGGACGAAAGTCTGATGGAGCAACGCCGCGTGAACGATGAAGGTCTTCGGATTGTAAAGTTCTGTTGTTAGGGACGAATAAGTACCGTTCGAATAGGGCGGTACCTTGACGGTACCTGACGAGAAAGCCACGGCTAACTACGTGCCAGCAGCCGCGGTAATACGTAGGTGGCAAGCGTTGTCCGGATTTATTGGGCGTAAAGCGCGCGCAGGCGGCTATGTAAGTCTGGTGTTAAAGCCCGGGGCTCAACCCCGGTTCGCATCGGAAACTGTGTAGCTTGAGTGCAGAAGAGGAAAGCGGTATTCCACGTGTAGCGGTGAAATGCGTAGAGATGTGGAGGAACACCAGTGGCGAAGGCGGCTTTCTGGTCTGTAACTGACGCTGAGGCGCGAAAGCGTGGGGAGCAAACAGGATTAGATACCCTGGTAGTCCACGCCGTAAACGATGAGTGCTAGGTGTTGGGGGTTTCAATACCCTCAGTGCCGCAGCTAACGCAATAAGCACTCCGCCTGGGGAGTACGCTCGCAAGAGTGAAACTCAAAGGAATTGACGGGGGCCCGCACAAGCGGTGGAGCATGTGGTTTAATTCGAAGCAACGCGAAGAACCTTACCAGGTCTTGACATCCCGCTGACCGCTCTGGAGACAGAGCTTCCCTTCGGGGCAGCGGTGACAGGTGGTGCATGGTTGTCGTCAGCTCGTGTCGTGAGATGTTGGGTTAAGTCCCGCAACGAGCGCAACCCTTATCTTTAGTTGCCAGCATTCAGTTGGGCACTCTAGAGAGACTGCCGTCGACAAGACGGAGGAAGGCGGGGATGACGTCAAATCATCATGCCCCTTATGACCTGGGCTACACACGTGCTACAATGGTTGGTACAACGGGATGCTACCTCGCGAGAGGACGCCAATCTCTTAAAACCAATCTCAGTTCGGATTGTAGGCTGCAACTCGCCTACATGAAGTCGGAATCGCTAGTAATCGCGGATCAGCATGCCGCGGTGAATACGTTCCCGGGCCTTGTACACACCGCCCGTCACACCACGGGAGTTTGCAACACCCGAAGTCGGTGAGGTAACCGCAAGGAGCCAGCCGCCGAAGGTGGAGAGTAGG

TR-4 16s sequence
